# TiO_2_-coated nanostructures for dye photo-degradation in water

**DOI:** 10.1186/1556-276X-9-458

**Published:** 2014-09-02

**Authors:** Viviana Scuderi, Giuliana Impellizzeri, Lucia Romano, Mario Scuderi, Giuseppe Nicotra, Kristin Bergum, Alessia Irrera, Bengt G Svensson, Vittorio Privitera

**Affiliations:** 1 CNR-IMM MATIS, Via S. Sofia 64, Catania 95123, Italy; 2 Department of Physics and Astronomy, University of Catania, Via S. Sofia 64, Catania 95123 Italy; 3 CNR-IMM, Z.I. VIII Strada 5, Catania 95121, Italy; 4 Centre for Materials Science and Nanotechnology, Department of Physics, University of Oslo, Blindern, P.O. Box 1048, Oslo 0318, Norway; 5 CNR-IPCF, Viale F. Stagno d'Alcontres 37, Faro Superiore, Messina 98158, Italy

**Keywords:** TiO_2_, Nanostructure, Photocatalysis, Water, 81.07.-b, 81.15.Gh, 89.60.Ec

## Abstract

The photocatalytic efficiency of a thin-film TiO_2_-coated nanostructured template is studied by dye degradation in water. The nanostructured template was synthesized by metal-assisted wet etching of Si and used as substrate for the deposition of a thin film of TiO_2_ (10 nm thick) by atomic layer deposition. A complete structural characterization was made by scanning and transmission electron microscopies. The significant photocatalytic performance was evaluated by the degradation of two dyes in water: methylene blue and methyl orange. The relevance of the reported results is discussed, opening the route toward the application of the synthesized nanostructured TiO_2_ for water purification.

## Background

Water purification has become a worldwide problem, in particular in industrialized countries, where wastewaters usually contain organic pollutants, such as dyes from the textile industry, leather tanning industry, paper production, food technology, agricultural research, pharmaceutical industry, etc. [1]. Due to their large-scale production and extensive applications, the organic dyes have become an important part of industrial wastewaters. Indeed, of the 7 × 10^5^ tons, more than 10% to 15% is lost in the wastewaters during the manufacturing and application processes [2]. The discharge of these colored compounds in the environment raises much concern because of the toxic effects on the ecological systems. Among others, two families of dyes - azo dyes and thiazine dyes - can cause serious health risk factors (see, for examples, refs. [3] and [4], respectively). It is also well known that some azo dyes are highly carcinogenic [5]. Since conventional wastewater treatment plants cannot degrade the majority of these pollutants, powerful methods for the decontamination of dyes in wastewaters have received increasing attention over the past decade.

Semiconductor photocatalysts have shown a great potential in water purification [6-8]. Among them, TiO_2_ (commonly called ‘titania’) is one of the most studied due to its unique characteristics: non-toxicity, good chemical stability, strong mechanical properties, low cost, and excellent photocatalytic performance [9]. The mechanism behind TiO_2_ photocatalysis has been deeply investigated: (1) electron-hole pairs are photo-generated upon bandgap excitation (3.15 eV for the anatase phase, 3.05 eV for the rutile crystalline phase [10]); (2) depending on the excitation lifetime relative to that of charge recombination, a net fraction of photo-generated charges can reach the surface; (3) the photo-generated charges can react with water by producing radical species, such as OH·, which are directly involved in the oxidation processes of water pollutants. TiO_2_ nanostructures can offer advantages such as high surface-area-to-volume ratio, enhancing in this way the amount of the photo-generated charges. TiO_2_ nanoparticles have been largely tested and demonstrated successful results [11]. However, there are some issues that strongly limit their application: poor light penetration due to nanoparticle agglomeration and the post-recovery of the particles after the water treatment [8]. An alternative to suspension is the thin film system where the photocatalyst is present as a thin film on the reactor walls [12], and recent investigations are oriented toward photocatalyst immobilization [7,8]. This kind of reactor promotes light penetration, and the coated area may be increased by packing with a material coated with the photocatalyst. A recent work experimentally quantified the charge diffusion length in high-quality titania: 3.2 nm for the anatase phase and 1.6 nm for the rutile phase, showing that a surface region of a few-nanometer depth provides charge carriers for photoreactions [13]. This clearly means that the use of a thick titania is useless.

Based on the above-mentioned considerations, we studied the photocatalytic activity of a TiO_2_ thin film covering a nanostructured Si template in degrading dyes in water. The titania film (10 nm thick) was obtained by atomic layer deposition (ALD). The ALD technique provided the possibility to efficiently enhance the exposed surface of the TiO_2_ since it offers an excellent conformality on high-aspect-ratio structures, as well as a great thickness control at atomic level [14]. The ALD was already used to create thicker (>30 nm) nanostructured TiO_2_, starting from nanotemplates [15,16]. Of course, thinner layers avoid a waste of material and enhance the nanostructuring effect. It is worth noting that highly anisotropic nanostructures such as nanotubes, nanorods, nanowires, and nanoribbons have been explored, but it is hard to compare the data from the literature in order to disentangle the real effect of the surface/volume enhancement from other contributions because of the complexity of the photocatalysis mechanism and the delicacy of the characterization techniques [12]. For example, most nanostructures are polycrystalline and the effect of grain boundaries and structural defects on charge transport cannot be neglected, especially when highlighting the beneficial effect of a certain photocatalyst shape over another one. Therefore, it is relevant to test the photocatalytic properties on a nanostructured material that has a reference with the same structural and compositional properties in a flat shape. In our experiment, the ALD provided a perfect tool to produce a reference flat sample with the same characteristics of the nanostructured sample.

## Methods

Nanostructured Si templates were obtained starting from p-type (10^16^ B/cm^3^), (100) Si wafers. The samples were UV oxidized and dipped in a 5% HF solution so as to obtain a clean and oxide-free Si surface. Then a thin Au layer (2 nm) was deposited on Si at room temperature by electron beam evaporation by using high-purity (99.9%) gold pellets as source. Finally, the samples were etched at room temperature in a solution of HF (5 M) and H_2_O_2_ (0.44 M) [17]. The templates were covered with a thin layer of TiO_2_ (10 nm thick), deposited by ALD, using a Beneq TFS 200 system (Beneq Oy, Espoo, Finland), with TiCl_4_ (99.9%) and de-ionized water as precursors, at a deposition temperature of 200°C. Nitrogen (>99.999%) was used as carrier gas. This sample typology will be hereafter called ‘TiO_2_/Si-template’. TiO_2_ flat films (10 nm thick) deposited on flat Si substrates were used as a reference, hereafter simply called ‘TiO_2_’.

The structural characterization was performed by scanning electron microscopy (SEM) with a field emission Zeiss Supra 25 (Carl Zeiss, Inc., Oberkochen, Germany) and by transmission electron microscopy (TEM) with a JEOL JEM-2010 F (JEOL Ltd., Akishima-shi, Japan) operated at 200 keV and equipped with a post-column Gatan GIF 2001 energy image filter (Gatan, Inc., Pleasanton, CA, USA).

The photocatalytic activity of the investigated materials was tested by the degradation of two dyes: methylene blue (MB) and methyl orange (MO), complying with the ISO protocol [18]. The employed MB was a 0.05-wt% solution in water (code number: 319112, by Sigma-Aldrich Corporation, St. Louis, MO, USA), while the MO was a 0.1% solution (code number: 34576, by Sigma-Aldrich Corporation, St. Louis, MO, USA). The irradiation was performed with a polychromatic UV lamp (from 350 to 400 nm), with a power of 8 W (by Philips, Amsterdam, The Netherlands). Before any measurement, the samples were irradiated by the UV lamp for 50 min in order to remove the hydrocarbons from the sample surface [19]. The samples, 0.6 cm × 0.8 cm in size, were immersed in a solution (2 ml) containing MB or MO and de-ionized water (starting concentration 1.5 × 10^−5^ or 1 × 10^−5^ M, respectively). The mixture was irradiated by the UV lamp with an irradiance of 1.1 mW/cm^2^. The irradiated solution was measured at regular time intervals with an UV-VIS spectrophotometer (PerkinElmer Lambda 35, PerkinElmer, Waltham, MA, USA) in a wavelength range from 500 to 800 nm for MB and from 350 to 650 nm for MO. The degradation of MB and MO was evaluated by the absorbance peak at 664 and 464 nm, respectively, in the Lambert-Beer regime [20]. The decomposition of the colorants in the absence of any catalyst materials was checked as a reference. Control experiments in the dark were conducted to clarify the contribution of the adsorption of the MB and MO at the sample surface.

## Results and discussion

**Figure 1:**
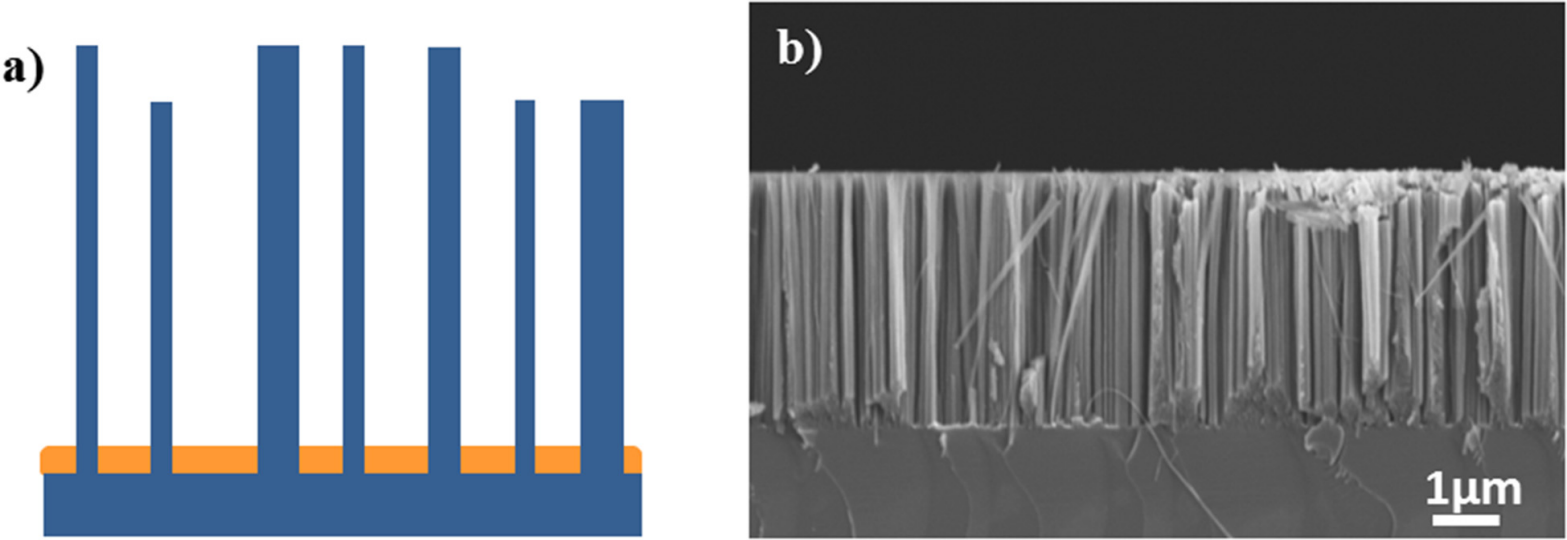
**Scheme and SEM image of the nanostructured Si template. (a)** Scheme of the nanostructured Si template (the Si is indicated in blue and Au in orange) and **(b)** the relative SEM image in cross-view.

The scheme of the nanostructured Si template is reported in Figure 1a in cross view, where the Si is indicated in blue and Au in orange. Figure 1b shows a cross-view SEM image of the template, which is formed by pillars approximately 4 μm long.

**Figure 2:**
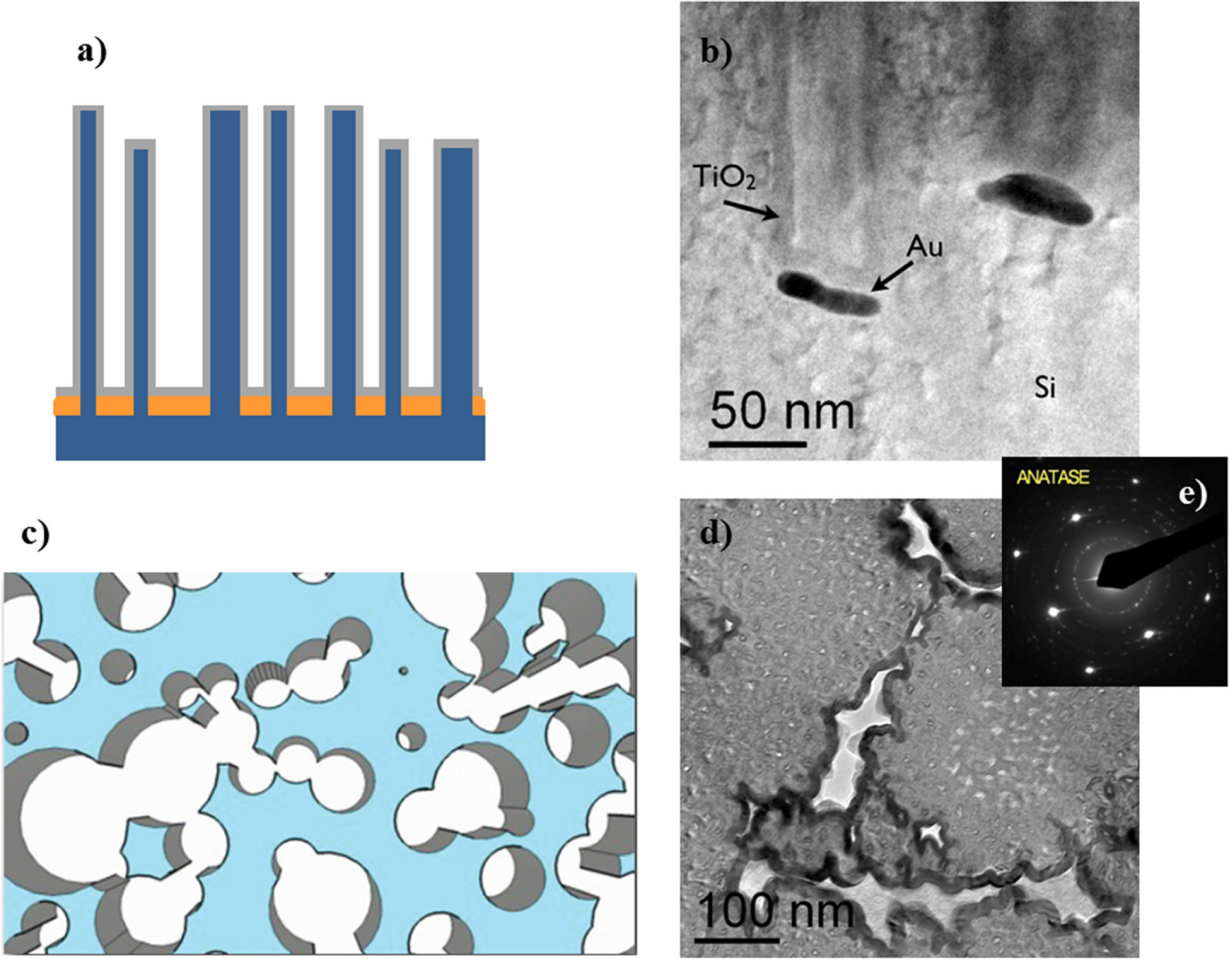
**Schemes and TEM images of the nanostructured Si template covered by the TiO**_**2 **_**and its diffraction pattern. (a)** Scheme of the nanostructured Si template after the TiO_2_ deposition and **(b)** the relative TEM image in cross-view. **(c)** Scheme of the sample after the TiO_2_ deposition and **(d)** the relative TEM image in plan-view. **(e)** Diffraction pattern showing silicon and polycrystalline TiO_2_.

The scheme of the nanostructured material after the deposition of the TiO_2_ layer is shown in Figure 2a in cross view, where the TiO_2_ is indicated in gray. A cross-view TEM image of the structure is shown in Figure 2b. The micrograph exhibits the Si substrate at the bottom of the structure; the Au nanoparticles involved in the wet etching process are visible in dark contrast; the top of the Au nanoparticles and the Si structures resulted to be uniformly covered by the TiO_2_ layer (10 nm thick). The analyses confirmed the excellent conformality of the deposition, thanks to the good diffusion of the precursors inside the nanostructured template, so the TiO_2_ coverage came up to the bottom of the Si template, despite the high aspect ratio of the nanostructures (approximately 100). Figure 2c shows a schematic plan-view of the sample in order to give a visual idea of the template structure with nanocavities, while Figure 2d reports the relative TEM image. Here, the light area indicates the nanocavities of the porous structure, while the dark gray area indicates the Si covered by the titania layer. A 100% enhancement of the TiO_2_ exposed surface area with respect to the flat film has been calculated by using the TEM data from several images similar to Figure 2d, thanks to the Gatan Digital Micrograph program. The diffraction pattern reported in Figure 2e unequivocally showed a polycrystalline anatase phase of the TiO_2_, in good agreement with the literature [21]. X-ray diffraction analyses indicated an average grain size of approximately 15 nm. The polycrystalline structure of the titania films resulted to be the same for both the TiO_2_/Si-template and the TiO_2_ flat sample.

**Figure 3:**
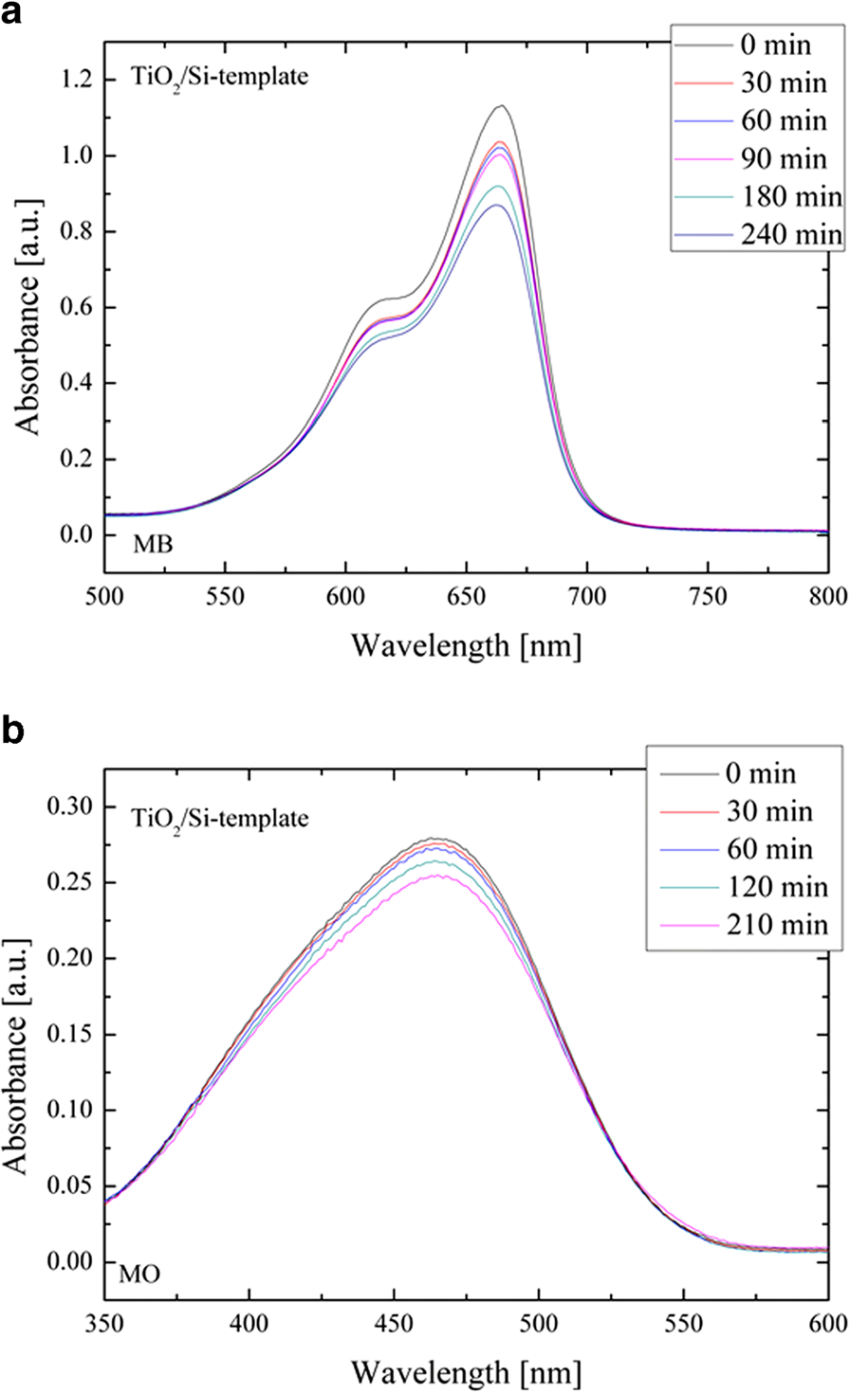
**Absorption spectra for (a) MB and (b) MO solutions for different irradiation times for the TiO**_**2**_**/Si-template samples.**

The photocatalytic activity of the synthesized materials was tested by the degradation of two dyes: MB, which is a dye of the thiazine family, and MO, which is a dye of the azo family (about the toxicity effects of these two dye families, the reader can refer to the ‘Background’ section). Figure 3 illustrates the discoloration measurements. In more detail, it reports the absorption spectra for the MB and MO (Figure 3a,b, respectively) solutions for different irradiation times for the TiO_2_/Si-template samples. The absorbance peaks at 664 and 464 nm are a direct measurement of the MB and MO concentrations, respectively (through the Lambert-Beer law [20]), and thus, their decrease with the UV irradiation time is a measure of the photocatalytic decomposition of the MB and MO molecules. The absence of any new absorption bands is indicative of the absence of by-product formation during the dye degradation processes [22].

**Figure 4:**
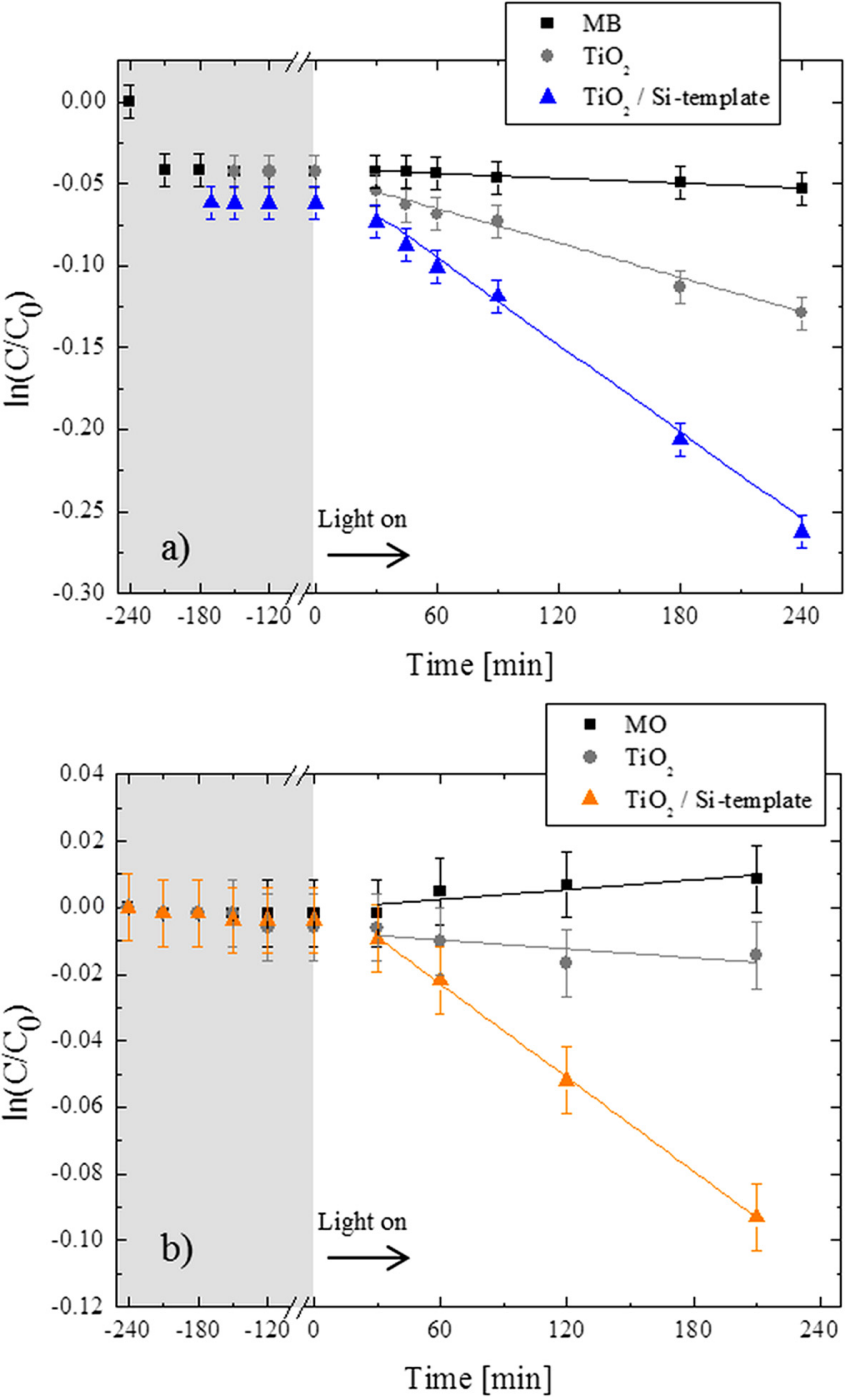
**MB and MO degradation for the three samples. (a)** MB and **(b)** MO degradation for the three samples: the solution (squares), the solution with the TiO_2_ flat film (circles), and the solution with the TiO_2_/Si-template sample (triangles). Measurements in the dark are indicated with the gray-colored region, while the ones under the UV irradiation are indicated with the white-colored region. The linear fits of the experimental data are indicated with the continuous lines.

The residual concentrations (ln(*C*/*C*_0_)) of the MB and MO dyes are reported in Figure 4a,b, respectively (*C* is the concentration of the organic species, *C*_0_ is the starting concentration of the organic species). Three samples were tested: the solution (MB or MO in de-ionized water) in the absence of any catalyst (squares), the solution with the TiO_2_ flat film (circles), and the solution with the TiO_2_/Si-template (triangles). The solution was first kept in the dark (from −240 min); at −180 min, the sample was immersed and kept in the dark (up to 0 min). The results reported in Figure 4a,b (gray-colored region) clearly show that there is a clear effect of the MB adsorption at the beaker walls in the absence of any catalyst materials (squares in Figure 4) in the first 30 min. This is not observed for the MO, probably due to the different nature of the two dyes: the MB is a cationic dye, while the MO is an anionic dye. The adsorption at the material surface in the dark is mainly negligible (circles and triangles in Figure 4), with the exception of a slight adsorption of the MB at the TiO_2_/Si-template surface during the first 10 min (square at −180 min and triangle at −170 min). Thus, the efficiency of the nanostructured TiO_2_ in degrading the dyes under the UV irradiation can be exclusively attributed to the photocatalytic effects. Figure 4 shows that the TiO_2_/Si-template exhibits the greatest dye degradation. According to the Langmuir-Hinshelwood model, the photo-degradation reaction rate, *k*, of water contaminants is given by the following reaction:

(1) $$\ln\left(\frac{C}{C_{0}}\right)=-\mathrm{kt},$$

where *C* is the concentration of the organic species, *C*_0_ is the starting concentration of the organic species, and *t* is the irradiation time [8]. By fitting the experimental data (lines in Figure 4) with Equation 1, the reaction rate for the MB degradation resulted to be 9.0 × 10^−4^ min^−1^ for the TiO_2_/Si-template, which is approximately three times higher than the reaction rate of the TiO_2_ flat film (3.6 × 10^−4^ min^−1^).

In order to evaluate whether the photocatalytic process might be limited by the diffusion process in water of the MB into the holes, we considered the diffusivity of MB in water of approximately 10^−8^ cm^2^/s [23]. Assuming that this value can be applied also in our porous structure, it would give a diffusion time to reach the bottom of the nanostructured sample (few microns) of few seconds. Therefore, in the time scale of this experiment, the photocatalytic process is not diffusion limited. Furthermore, considering the slight adsorption of the MB at the TiO_2_/Si-template surface during the first 10 min (square at −180 min and triangle at −170 min), we directly measured the adsorption rate (by Equation 1), which resulted to be 3.0 × 10^−3^ min^−1^, which is about three times higher than the reaction rate for the MB degradation, clearly demonstrating that the adsorption process is not limiting the photocatalytic one.

The reaction rate for the MO degradation resulted to be 4.7 × 10^−4^ min^−1^ for the TiO_2_/Si-template, which is approximately 12 times higher than the reaction rate of the TiO_2_ flat film (4.0 × 10^−5^ min^−1^).

The synthesized material showed the highest degradation rate in the case of the MB. The observed difference between the MB and MO degradation efficiencies is not surprising, since it is well assessed that it is not possible to realize the *best* photocatalyst, but every TiO_2_ material is able to efficiently degrade an organic compound, but less efficiently another one, due to the various parameters governing the photocatalytic reactions [24].

The marked difference in the photocatalytic response between the TiO_2_ flat sample and the TiO_2_/Si-template can be explained by taking into account the observed 100% enhancement of the TiO_2_ exposed surface area with respect to the flat film. A quantitative comparison between the exposed surface area enhancement and the dye discoloration would not be a rigorous method because (1) the calculated enhancement is an underestimation, since with the used field of view of the microscopy images, there was a limit in the visibility of the holes with a diameter smaller than approximately 4 nm, and (2) the photocatalysis mechanism is complex.

The possible contribution of the Au nanoparticles in the photocatalytic activity of TiO_2_[25] can be excluded since the surface of gold is negligible with respect to the exposed surface of the TiO_2_/Si-template (approximately 100 times less than the titania exposed surface). In addition, since the charge diffusion length in high-quality titania has been reported to be 3.2 nm for the anatase phase [13], and since the TiO_2_ ALD layer reported in this work is 10 nm thick, we can exclude any contribution of the Au nanoparticles, placed underneath the TiO_2_ layer. The same argument can be applied in order to exclude the possible effect of the Si support on the photocatalytic activity of the nanostructured TiO_2_.

## Conclusions

In conclusion, we synthesized nanostructured TiO_2_ with the main aim of enhancing the photocatalytic activity through an increase of the exposed surface area. The material was synthesized by using a nanostructured Si template obtained by metal-assisted wet etching of Si substrates. The realized template was covered with a thin layer of TiO_2_ (10 nm thick), deposited by ALD. This approach avoided the use of nanoparticles and their consequent dispersion in water. The reported results show that the excellent conformality of the titania film on high-aspect-ratio Si nanostructures is responsible for the improved efficiency in degrading dyes in water. In particular, the nanostructured TiO_2_ exhibited a photo-degradation reaction rate for the MB and MO that is approximately 3 and approximately 12 times the rate of the TiO_2_ flat film, respectively. Thus, our results demonstrate that the TiO_2_ thin film coating of nanostructured surface can be efficiently used for water treatment reactors.

## Competing interests

The authors declare that they have no competing interests.

## Authors’ contributions

VS performed the photo-degradation measurements and wrote the paper. GI supervised all the experiments, interpreted the data, and wrote the paper. LR conceived the study and performed the SEM analyses. MS and GN carried out and interpreted the TEM analyses. KB and BGS performed the ALD deposition. AI synthesized the nanostructured Si template. VP supervised the whole project. All authors read and approved the final manuscript.
